# Duration of an intense laser pulse can determine the breakage of multiple chemical bonds

**DOI:** 10.1038/srep12877

**Published:** 2015-08-14

**Authors:** Xinhua Xie, Erik Lötstedt, Stefan Roither, Markus Schöffler, Daniil Kartashov, Katsumi Midorikawa, Andrius Baltuška, Kaoru Yamanouchi, Markus Kitzler

**Affiliations:** 1 Photonics Institute, Vienna University of Technology, Gusshausstrasse 27, A-1040 Vienna, Austria, EU; 2 Laser Technology Laboratory, RIKEN, 2-1 Hirosawa, Wako, Saitama 351-0198, Japan; 3 RIKEN Center for Advanced Photonics, 2-1 Hirosawa, Wako, Saitama 351-0198, Japan; 4 Department of Chemistry, School of Science, The University of Tokyo, 7-3-1 Hongo, Bunkyo-ku, Tokyo 113-0033, Japan

## Abstract

Control over the breakage of a certain chemical bond in a molecule by an ultrashort laser pulse has been considered for decades. With the availability of intense non-resonant laser fields it became possible to pre-determine femtosecond to picosecond molecular bond breakage dynamics by controlled distortions of the electronic molecular system on sub-femtosecond time scales using field-sensitive processes such as strong-field ionization or excitation. So far, all successful demonstrations in this area considered only fragmentation reactions, where only one bond is broken and the molecule is split into merely two moieties. Here, using ethylene (C_2_H_4_) as an example, we experimentally investigate whether complex fragmentation reactions that involve the breakage of more than one chemical bond can be influenced by parameters of an ultrashort intense laser pulse. We show that the dynamics of removing three electrons by strong-field ionization determines the ratio of fragmentation of the molecular trication into two respectively three moieties. We observe a relative increase of two-body fragmentations with the laser pulse duration by almost an order of magnitude. Supported by quantum chemical simulations we explain our experimental results by the interplay between the dynamics of electron removal and nuclear motion.

Manipulation of physical or chemical processes and control over their evolution, e.g., the breaking of a certain chemical bond in a polyatomic molecule, has been acknowledged as a crucial ability in both science and technology for decades. The nuclear motion involved in the breaking of a chemical bond in a molecule typically proceeds on time scales from several femtoseconds to picoseconds. These dynamics are driven by the derivatives of the potential formed by the intra-molecular electron distribution, which can restructure on much faster, attosecond, time scales. A suitable perturbation of the equilibrium bound electronic distribution, for example induced by ultrashort intense laser pulses, can therefore initiate nuclear motion towards a desired bond-breaking event. A relatively slow molecular fragmentation can thus be pre-determined on the much faster electronic time scale. This has been demonstrated for fragmentation reactions resulting in two moieties using the carrier-envelope offset phase of few-cycle laser pulses as the control parameter^1^,^2^,^3^,^4^,^5^,^6^,^7^,^8^,^9^,^10^, and by selective removal of electrons from either inner or outer valence orbitals based on their different shapes^11^ or their different sensitivity to laser intensity and/or pulse duration^12^.

In this paper we investigate whether molecular fragmentation reactions into three moieties that involve the breakage of two chemical bonds can be determined during the interaction with an ultrashort intense laser pulse. The implications of this extension by one moiety are vast, and a number of different processes become possible that are not present for two-body fragmentations. For example, a three-body fragmentation, which can only occur in a polyatomic molecules, takes place along (at least) two nuclear coordinates. Thus, the fragmentation dynamics must be necessarily described by molecular potential energy surfaces (PESs) rather than only one-dimensional potential energy curves. Furthermore, if a polyatomic molecule fragments into three moieties, the sequence and timing of the two involved fragmentation steps become important^13^: The two fragmentation steps can occur concertedly (simultaneous breaking of two bonds) or sequentially (one after another). For a sequential fragmentation dynamics it becomes additionally important which one of the two involved bonds breaks first.

A very interesting question is then, whether it is possible to use a specific parameter of a strong laser field for determining the bond-breaking dynamics during a three body-fragmentation reaction of a polyatomic molecule, in particular the branching ratio between different fragmentation channels. This question has been investigated for various molecules and different laser parameters, e.g., intensity and polarization state^13^ or chirp-rate^14^,^15^. As the laser-driven fragmentation of a polyatomic molecule is dictated by the relative dynamics of the nuclei and the electrons, as well as the coupling between these two dynamical systems, the duration of the driving laser pulse is a crucial parameter. This point has been investigated, e.g., by experiments^16^,^17^,^18^ and simulations^19^,^20^ using the simplest polyatomic molecules, 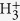 /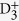 and isotopic variants, as model systems. It has been shown that due to the different nuclear dynamics, isotopic variants exhibit different dependence of the branching ratio of certain channels on the pulse duration^18^.

Here, using the example of three-body fragmentation of the much more complicated ethylene molecule, C_2_H_4_, we demonstrate experimentally that the pulse duration of strong laser pulses can determine with a high confidence the relative probability whether the molecule fragments into three respectively two moieties, i.e., whether two bonds or only one bond breaks in the molecule. Our experiments thus show that a suitable choice of the laser pulse duration can determine the ratio of molecules within an ensemble of identical neutral ethylene molecules that fragment into two or three moieties, respectively. In contrast, in the abovementioned experiments^18^ the preponderance towards fragmentation into a certain channel is introduced by the different dynamics of different isotopic variants when exposed to shorter or longer pulses. However, data taken for two pulse durations indicate a sensitivity of the relative yields of two-body and three-body fragmentation channels of 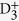 on the pulse length^17^, although this observation was not further investigated.

We will show below that the underlying mechanism for the capability of determining the multi-particle fragmentation dynamics of ethylene builds on the field-induced dynamical distortions of the bound electron density, in particular the dynamics of removing three electrons by strong-field ionization: Squeezing the removal of three electrons to a very short period of less than two optical cycles (≲5fs), results in an almost exclusive decomposition of the molecules into three fragment ions. However, if the three electrons are allowed to be ejected during a longer period (≳12fs), the relative probability that the molecules decompose into only two ionic fragments increases by roughly an order of magnitude, while at the same time the relative probability of three-body fragmentation decreases accordingly. Supported by quantum chemical simulations we explain our experimental results by the coupling between the electronic and nuclear degrees of freedom, specifically the interplay between the dynamics of electron removal and nuclear motion, in particular C-H bond stretching.

## Results

In our experiments we measured in coincidence the three-dimensional (3D) momentum vectors of fragment ions resulting from the interaction of an ultrashort intense laser pulse with an ethylene molecule using coincidence momentum imaging^21^. Details on the experimental setup are provided in Methods. Imposing coincidence conditions on the measured data, as described in Methods, we identified for all laser pulse parameters used in the experiments two fragmentation channels where the ethylene trication decomposes into two ionic fragments,


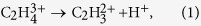



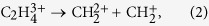


and two channels where three fragment ions are produced,


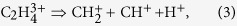



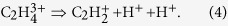


The double arrows in Eqs (3) and (4) shall indicate that the final fragmentation products on their right hand side can be produced by various fragmentation dynamics, which will be investigated and described in detail in the following. The trication itself was not detected in our experiments, which indicates that all states in this species that were populated during the laser-interaction are dissociative.

### Fragmentation dynamics

**Figure 1:**
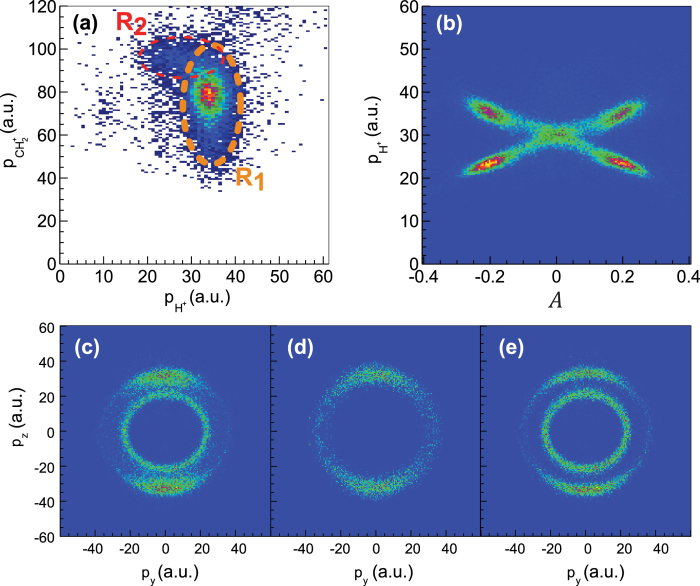
(**a**) Momentum correlation map for the final products 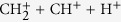. (**b**) Fragmentation yield over proton momentum and the asymmetry parameter *A* as defined in the text for the final products 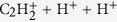. (**c**) Momentum distribution in the laser polarization plane for the reaction shown in (**b**). (**d,e**) Proton spectra in the laser polarization plane decomposed from (**c**) based on the asymmetry parameter *A*. The proton spectra ejected via the concerted (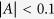) and sequential (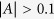) fragmentation pathway are shown (**d**) and (**e**), respectively. The laser pulse duration and peak intensity are 25 fs and 8 × 10^14^W/cm^2^ for all panels. Here and throughout the paper a.u. denotes atomic units.

As mentioned above, the dynamics of a three-body fragmentation reaction can take place concertedly or sequentially. Conclusive insight into the dynamics can be gained from momentum correlation maps^13^,^22^. Such a map is depicted in Fig. 1(a) for the channel (3). It shows the absolute values of the momenta of the two fragments H^+^ and 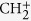 with the momentum of the third fragment, CH^+^, integrated over. The map exhibits two distinct regions. In region *R*_1_ the proton momentum is largely independent of that of the fragment 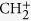. This indicates that the two fragments are generated during two independent fragmentation steps. Also the proton exhibits high momentum (corresponding to a mean energy of about 8.6 eV), which implies that it is ejected from a high charge state. Thus, region *R*_1_ corresponds to a sequential fragmentation reaction, during which a proton is ejected during the first fragmentation step, see Eq. (5)


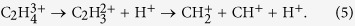


In region *R*_2_ the momentum of 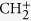 is largely independent of that of the proton, indicating that these two fragments are produced during two different fragmentation steps. Also the proton momentum is smaller. Thus, fragmentation events in region *R*_2_ correspond to a sequential fragmentation reaction, in which the proton is ejected during the second step, see Eq. (6)


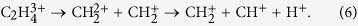


Clarification of the fragmentation dynamics of channel (4), which consists in the production of two protons, is more difficult but possible by defining an asymmetry parameter *A* = (*p*_1_−*p*_2_)/(*p*_1_ + *p*_2_), where *p*_1_ and *p*_2_ denote the absolute values of the momenta of the two protons. Plotting the measured proton momenta over *A*, see Fig. 1(b), reveals an x-shaped structure. The center point of the structure, 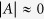, corresponds to protons with equal momenta ejected from the same charge state. Thus, fragmentations in the region |*A*| < 0.1 take place concertedly, see Eq. (7)


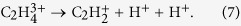


Outside the center region the two protons have distinctively different momenta and asymmetry, thus, they are generated during different fragmentation steps according to Eq. (8)
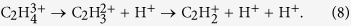


The confidence of deciding whether the fragmentations take place via the reaction Eq. (7) or Eq. (8), respectively, using the asymmetry parameter *A* as a criterion is very high. Only artificially constructed situations that involve, for example, fast charge migration processes during the two fragmentation steps, or still more unlikely extremely fast nuclear motion such as a precisely synchronized asymmetric C-H stretch motion, can lead to 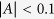 for a sequential fragmentation dynamics. For all reasonable situations we can be sure that 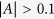 corresponds to a sequential process. Based on a selection in *A* it is therefore possible with a high confidence to precisely decompose the overall proton momentum distribution in the laser polarization plane, shown in Fig. 1(c), into that corresponding to the concerted fragmentation dynamics, Fig. 1(d), and the one corresponding to a sequential dynamics with its clearly defined two different proton energies, see Fig. 1(e).

### Pulse duration dependence of fragmentation dynamics

**Figure 2:**
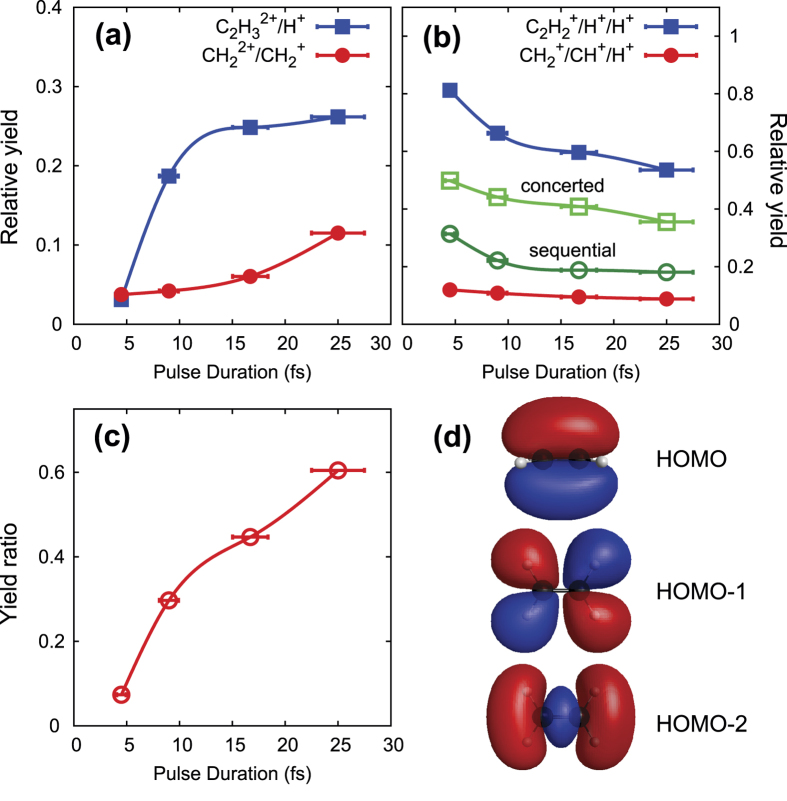
(**a**) Normalized yield of channels (1) and (2) as a function of laser pulse duration. (**b**) Normalized yield of channels (3) and (4) as a function of laser pulse duration. The yield of channel (4) is divided into that of the concerted (7) and sequential (8) fragmentation dynamics, respectively. Normalization is such that the sum of the yields of channels (1) to (4) is 1 at each pulse duration. (**c**) Ratio of two-body vs. three-body yield as a function of laser pulse duration. The laser peak intensity is 8 × 10^14^W/cm^2^ for all data points in all panels. All lines are only to guide the eye. (**d**) Schematics of molecular valence orbitals of neutral ethylene calculated by GAMESS^26^.

Having identified the dynamics of the fragmentation reactions that lead to the detected ionic moieties, we now turn to investigate the electronic origin of the separating motion of the molecular fragments. To this end we measured the relative yield of all four identified two-body and three-body fragmentation channels as a function of pulse duration from 4.5 fs to 25 fs (FWHM) with a constant peak intensity of 8 × 10^14^ W/cm^2^. The results of these measurements, depicted in Fig. 2(a,b), show that the probability of two-body fragmentation via breaking of a C-H bond [channel (1)] dramatically increases as the pulse duration is increased from 4.5 fs to about 12 fs, and levels off for longer pulse durations. We note, that the time scale of 12 fs is very close to the C-H vibrational period of about 11 fs for both the neutral^23^ and dication^24^.

As described in Methods, to increase the pulse duration the shortest pulses (4.5 fs) were stretched by dispersive propagation through suitable amounts of fused silica. Pulses with a duration of 25 fs were taken directly after the prism compressor of the laser amplifier system. Thus, we would like to note that the chirp of the laser pulses stretched to 9 fs and 17 fs, respectively, could, in principle, influence the measured yield at the corresponding two data points in Fig. 2(a,b)^14^,^15^. However, it can be seen, especially in Fig. 2(b), that these two data points nicely follow the overall trend of the data from 4.5 to 25 fs. From that we conclude that even if the chirp somewhat influences the exact value of the yield, it surely does not qualitatively change the overall effect of increasing the pulse duration. We will come back to the more fundamental reasons for the apparent insensitivity of the yield on the chirp in the discussion section.

Figure 2(a) furthermore shows that also the relative yield of two-body fragmentation via breaking of the center C-C bond monotonically increases with pulse duration. However, the slope is much smaller than in the case of the C-H bond, and the yield is still increasing at the longest measured pulse duration. We relate this to the slower fragmentation dynamics of the C-C bond, whose vibrational period is about 21 fs for the neutral^23^ and 38 fs for the dication^24^. In contrast to the two-body channels, the relative yields of the three-body fragmentation channels, for both the concerted and sequential fragmentation reactions, decrease with pulse duration [Fig. 2(b)]. A direct comparison of the cumulative two-body vs. the three-body fragmentation yield in Fig. 2(c) shows that the relative probability of the triply charged molecular ion to decompose into two fragments rather than three increases by roughly an order of magnitude when the laser pulse duration is increased from 4.5 to 25 fs.

### Ionization dynamics

To understand the molecular mechanisms behind this dramatic dependence of the fragmentation dynamics on the laser pulse duration, we first investigate which electrons are removed by the laser field during the three ionizations steps that lead to the molecular three-body breakup. To this end we analyze the angular distributions of the fragment ions. As discussed in detail in the Supplementary Information we find that the fragmentations are triggered by ionization events that involve the removal of electrons from orbitals that exhibit a charge density distribution peaking along the fragmentation direction, i.e. along the C-H and C-C bonds. As at the laser peak intensity used for the measurements in Fig. 2 ionization from the HOMO is already saturated and thus the ionization becomes insensitive to its shape^11^, we can conclude by comparison to Fig. 2(d) that during the second and third ionization steps significant electron removal takes place from HOMO-1 and HOMO-2. From this we infer that in the course of the three ionization steps the doubly charged ion will very likely be reached on an excited state that may be dissociative. This is an important conclusion which we will pick up on again below.

### Intra-molecular charge redistribution prior to fragmentation

Additional insight into the molecular dynamics that precede the fragmentation can be gained from the energy distributions of the fragment ions. These distributions contain information about the PESs on which the fragmentations take place and also on the molecular geometry and charge distribution right before the fragmentation event^12^,^13^,^25^. In the following we will focus on the dominant three-body fragmentation channel that consists of the ejection of two protons, which can take place concertedly, (7), or sequentially, (8).

**Figure 3:**
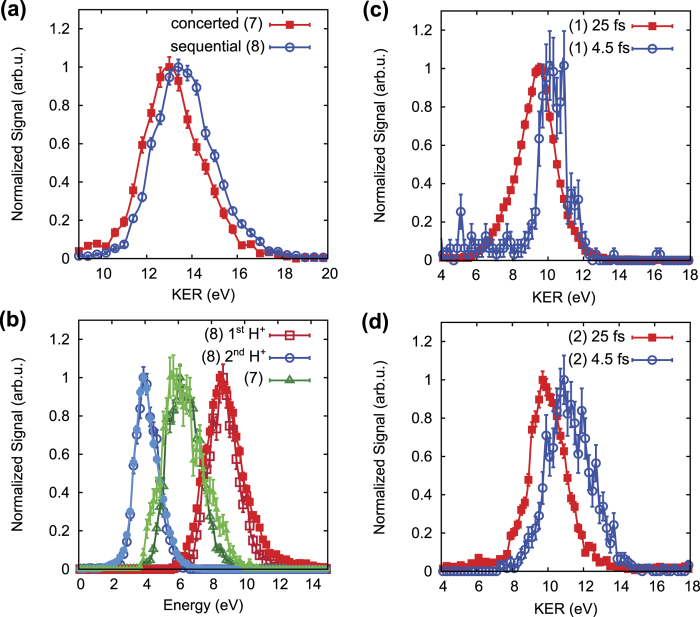
(**a**) Kinetic energy release (KER) distributions for the concerted (7) [red filled squares] and sequential fragmentation (8) [blue open circles] dynamics of fragmentation into the final products 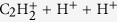. (**b**) Proton energy spectra for the concerted fragmentation reaction (7) [green triangles] and the first [red squares] and second [blue circles] proton ejected during the sequential fragmentation reaction (8) for a laser pulse duration of 25 fs [light full symbols] and 4.5 fs [dark open symbols]. (**c,d**) KER distributions for the two-body fragmentation reactions (1) [panel (**c**)] and (2) [panel (**d**)] for a laser pulse duration of 25 fs [red squares] and 4.5 fs [blue circles]. The laser peak intensity is 8×10^14^ W/cm^2^ for all data points in all panels.

Figure 3(a) shows the kinetic energy release (KER) distributions of these two fragmentation reactions. The KER of a certain fragmentation channel is defined as the sum of the kinetic energies of all fragment ions. Although the two reactions (7) and (8) feature the same final set of fragments, they show slightly different KER. The mean KER of the sequential process (8) is higher by 0.5 eV than the one of the concerted process (7). This may indicate that the initial intra-molecular charge distribution after triple ionization is different for these two processes. The following speculative scenarios can explain the observations: In the concerted process, prior to fragmentation, positive charge density needs to be situated on either of the two ejected protons. From the proton energy distributions in Fig. 3(b) we can infer that the remaining positive charge density is most probably situated in the center of the 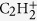 moiety, i.e. on the carbon skeleton structure. The latter statement can be understood by acknowledging that the energy distribution of both ejected protons is smooth and shows only a single peak. This means that both protons feel very similar Coulomb repulsion, and, hence, their distances to the repulsing positive charge need to be approximately equal prior to fragmentation.

In the sequential fragmentation reaction (8), in contrast, positive charge density needs to be situated on one of the protons prior to the first fragmentation step. The remaining positive charge density is most likely situated on the carbon structure in the center of the molecule. This can be seen from the higher kinetic energy of the proton ejected during the first fragmentation step, Fig. 3(b), that results from this higher charge concentration closer to the proton. After the ejection of the first proton, the positive charge density redistributes during the long life time of the remaining 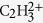 moiety that can be inferred from the angular momentum distributions (see Supplementary Information), and part of it may migrate to a second proton, while the rest stays in the center of the molecular ion. Fragmentation from this charge distribution then leads to a relatively low energy of the second ejected proton, see Fig. 3(b).

Because in the sequential process the positive charge density is initially distributed more concentratedly than in the concerted process, the KER is higher in the sequential process, as seen in Fig. 3(a). The fact that the initial intra-molecular charge distributions are different for the concerted and the sequential three-body fragmentation dynamics leads us to another important conclusion, namely that the two fragmentation reactions start from different PESs of the triply charged molecular ion.

### Ultrafast nuclear motion and field-induced excitations during molecular fragmentation

As described in the previous section, we could understand the different intra-molecular charge (re-)distribution dynamics underlying the concerted and sequential fragmentation reactions by combining the complementary information contained in the KER and proton energy distributions. Yet, the fragment energies are not only determined by the intra-molecular distribution of the holes, but also, and to a large extent, by the molecular structure, i.e. by the bond lengths. It has been shown that in particular the proton energies can change over a very wide range if the C-H bond lengths change during the laser pulse, which can take place as fast as on a sub-10 fs time scale^25^. We can, however, rule out C-H bond length variations as the source of the different KER values for the concerted and sequential fragmentation dynamics in Fig. 3(a) by acknowledging that the proton energies are independent of the laser pulse duration. This is shown in Fig. 3(b) by comparison of distributions measured with sub-5 fs and 25 fs laser pulses. A pulse duration of 25 fs is sufficiently long such that the C-H bonds could stretch significantly during the laser pulse^25^. A stretch of the C-H bond length would lead to a significant decrease of the proton energies. The striking similarity of the proton energy distributions for the two pulse durations, thus, signifies that the PESs from which the different versions of the three-body fragmentation reaction (4) start, are populated always at roughly the same coordinates, widely independent of the time span during which three electrons are removed.

We have furthermore measured the KER distributions of the channels (7) and (8) for different laser pulse durations in the range from 4.5 fs to 25 fs. No dependence of the KER of these two channels on the laser pulse duration was found (not shown in Fig. 3). As the fragmentation from a different (excited) state reached by multi-photon transitions may very likely lead to a different KER value^12^, and because the probability for field-driven excitations is much higher for multi-cycle than for few-cycle pulses, we may conclude from the similarity of the KER values for 4.5 fs and 25 fs that the probability of preparing the triply charged molecular ion in an electronically excited state by field-driven excitations is negligible.

In contrast to those of the three-body fragmentation processes, the KER of the two-body fragmentation reactions (1) and (2) significantly depend on the laser pulse duration, as can be seen in Fig. 3(c,d), respectively. The KER distribution of these two reactions shift to lower energies by about 1 eV when the laser pulse duration is increased from 4.5 fs to 25 fs. From this we may deduce that the respective bonds undergo ultrafast stretch dynamics in the course of the triple ionization, potentially after the first or second ionization event. For the shortest pulses all three ionization events happen within a very short time window and the molecular ion predominantly stays in the equilibrium geometry of neutral ethylene. For longer pulses, however, the C-H or C-C bonds, respectively, may already start to stretch after the first or second ionization event. This, consequently, leads to lower KER mean values.

## Discussion

We have shown above that the relative probability of triply charged ethylene to decompose into two rather than three moieties increases by almost one order of magnitude when the laser pulse duration, at constant peak intensity, is increased from 4.5 fs to 25 fs, see Fig. 2(c). This is the central finding of our work. To explain this dramatic change in fragmentation behavior we studied the fragment angular momentum distributions, KER and proton energy distributions for different pulse durations. By combining the complementary information contained in these distributions we found that

1. the doubly charged ion is prepared in an electronically excited state by lower-valence ionization,
2. significant stretch motion of the C-H and C-C bonds in the singly or doubly charged ion takes place for longer pulse durations,
3. field-driven excitations are unimportant for the three-body fragmentation reactions (7) and (8),
4. the respective initial C-H distances of the reactions (7) and (8) prior to fragmentation are independent of the laser pulse duration,
5. the concerted and sequential three-body fragmentation reactions, (7) and (8), take place on different PESs.

A scenario that can explain the dramatic increase of the relative two-body yield with pulse duration and that at the same time is in agreement with all these findings is the following one:

During the triple-ionization process the intermediate doubly charged ion is reached on an electronically excited PES as a result of removing (at least) one of the two electrons from an inner-valence shell. For sufficiently long laser pulse durations the C-H and C-C bonds do have enough time to significantly stretch on this excited PES. In contrast, for short pulse durations (4.5 fs) the nuclei stay essentially frozen. Thus, for short pulses the third ionization step prepares the triply charged molecular ion in a configuration close to the neutral’s equilibrium configuration. For longer pulses (25 fs), however, the triply charged molecular ion is reached with significantly stretched C-H and also slightly stretched C-C bonds. Thus, as we found that field-driven further excitation is insignificant, with longer pulses the triply charged ion is prepared energetically further down the dissociative PES, i.e., on a position with lower potential energy as compared to short pulses. As a consequence, for longer pulses the potential energy is too small to overcome the energy barrier towards triple fragmentation and only two-body fragmentation is possible. In effect, the two-body fragmentation probability becomes strongly enhanced with increasing pulse duration, as observed in the experiment [Fig. 2].

### Quantum chemical numerical verification

**Figure 4:**
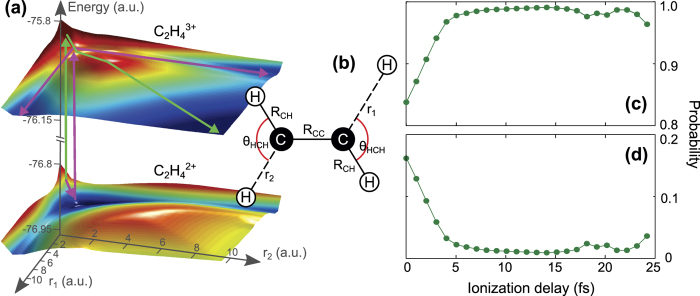
(**a**) Potential energy surfaces (PESs) in the ethylene dication (lower) and trication (upper) calculated by GAMESS as described in the text for the stretch motion of two C-H bonds marked by *r*_1_ and *r*_2_ in (**b**). (**b**) Schematics of the geometry used for the calculation of the PESs shown in (**a**). See text for details. (**c,d**) Simulated probability for fragmentation of 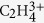 into two (**c**) and three (**d**) moieties along the reactions (1) and (7), respectively, as a function of delay between the second and third ionization step.

To verify the described scenario that follows from the experimental findings we performed simulations for the three-body fragmentation channel (4) using the quantum chemistry simulation package GAMESS^26^. We calculated the ground and several excited PES of the doubly and triply charged molecular ion for a stretch motion in the molecular plane of two opposing C-H bonds, see Fig. 4(b) for a schematics. The C-C bond length *R*_C*C*_ was fixed to *R*_C*C*_ = 2.49 a.u. the H—C—H angle *θ*_HCH_ was fixed to *θ*_HCH_ = 117°, and two C-H internuclear distances were set to *R*_CH_ = 2.03 a.u. These values correspond to the molecular geometry of neutral C_2_H_4_, as obtained from a geometry optimization performed with GAMESS at the Hartree-Fock (HF) level. For the calculation of the PESs, we used the 6-311G** basis set and the complete-active-space method, with two frozen core orbitals, and 10 active HF orbitals.

In agreement with the experimental data, which imply nuclear motion on an excited PES in the doubly charged ion, we find that all lower excited PESs exhibit a qualitatively similar shape with an C-H equilibrium distance larger than the neutral ethylene, i.e., they all feature pronounced C-H stretch motion. Thus, for obtaining a qualitative understanding of the three-body fragmentation dynamics it is sufficient to consider the first excited singlet *A″* state in the following discussion. At the equilibrium geometry of neutral C_2_H_4_ this state corresponds to removing one electron from the HOMO, and one electron from the HOMO-1 of the neutral ground state. The corresponding PES is shown as the lower PES in Fig. 4(a). Since the experiment shows that field-driven excitations are negligible, in our simulations we assume that the dominant three-body fragmentation reaction, i.e., the concerted reaction (7), takes place on the doublet *A′* ground state PES of the triply charged molecular ion, shown as the upper PES in Fig. 4(a). In the following we will focus onto this channel and ignore the less probable sequential fragmentation reaction (8) which, according to the above discussion, takes place on a different (excited) PES.

The two PESs in Fig. 4(a) confirm the fragmentation scenario described above. If the C-H bonds do not stretch in the doubly charged ion, the PES of the trication is reached sufficiently high up in potential energy such that the barrier towards concerted three-body fragmentation can be overcome. This is shown by the pathway marked by green arrows in Fig. 4(a), which applies to the case when ionization is performed by very short pulses. If the C-H bonds are given enough time to stretch by about 2.5 a.u. towards the equilibrium distance on a pathway marked by magenta arrows in Fig. 4(a), the PES of the triply charged ion is reached at a point that is too low in energy to overcome the barrier towards concerted three-body fragmentation. In that case, which applies to long pulse durations, fragmentation takes place via the stretch motion of only one of the two C-H bonds along the trench in the PES marked by the magenta arrows. Thus, the trication decomposes into only two rather than three fragments [channel (1)]. As the vibrational motion of the C-H bonds is fast (vibrational period about 11 fs, as noted above), the time that it takes for the C-H bonds to stretch by a small amount of 2.5 a.u. is only a few fs. Simulations that will be discussed below show that this stretch motion is completed within about 6 fs [Fig. 4(c,d)]. In other words, with pulses long enough such that the delay between the second and third ionization step can be on that order (or longer), the combined actions of this ionization-delay and the concomitant nuclear motion in the doubly charged ion guide the molecular trication to a local potential minimum from where two-body fragmentation is much more likely, cf. experimental data in Fig. 2.

The discussed scenario, that relies on the relative timing of the third ionization step to the C-H stretch dynamics, can also explain that the measured yield is apparently largely insensitive to the chirp of the laser pulses, as mentioned above. The reason is that, first, the C-H nuclear dynamics on the excited PES of the dication is largely unaffected by the field. Secondly, the trication is reached by strong-field ionization, which is also insensitive to relatively minor shifts of the instantaneous photon energy, as long as the ionization process is dominated by tunneling into the continuum rather than absorption of few photons, which for the present case of a large energy gap from the di- to the trication is fulfilled.

To confirm the timing of the nuclear motion assumed in the described scenario, we performed quantum dynamical simulations on the two PESs shown in Fig. 4(a) in order to calculate the dissociation probabilities for the considered two- and three-body reactions. Details about the simulations and about their limitations are provided in Methods. Because of the simplifications that we make in our model we cannot expect quantitative agreement with the experimental data. The aim of the simulations is therefore to qualitatively verify the scenario that we propose to explain our experimental results. The dissociation probabilities resulting from the simulations are shown in Fig. 4(c,d) as a function of the ionization delay time *τ*. The results of the simulation do reproduce qualitatively the trend of increasing (decreasing) two-body (three-body) yield with increasing delay time *τ*. Also the time scale of approximately 5 fs during which the two-body yield strongly increases is similar to the one measured in the experiment, cf. Fig. 2(a). The discrepancy with the experimental results may have several reasons. One of them might be the restriction to only two nuclear degrees of freedom. Another one might be the omission of the laser field during the wave packet propagation, which excludes dynamical distortions of the PESs^27^ that may influence the fragmentation dynamics.

## Conclusion

In conclusion we show, both experimentally and by quantum simulations, that the outcome of complex fragmentation reactions of the ethylene trication that involve the breakage of more than one chemical bond sensitively depends on the duration of the inducing intense, non-resonant, ultrashort laser pulse. Specifically we demonstrate that the ratio of yields for fragmentation into three vs. two fragment ions can be determined by using the duration of the laser pulses as a parameter. In our experiment, by increasing the laser pulse duration from 4.5 fs to 25 fs, the relative probability to fragment into two vs. three ionic fragments is enhanced by roughly an order of magnitude. We show that the underlying mechanism is the relative timing of successive electron release events and the concomitant nuclear motion: The longer period during which molecular bonds can stretch when interacting with long laser pulses, and the resulting lower potential energy available to the fragmentation, leads to a strong relative enhancement of the two-body fragmentation reaction. In contrast, for short pulses, for which the molecular bonds have almost no time to stretch, the molecular ion is prepared at higher potential energy and breakage of two bonds, i.e., fragmentation into three moieties, becomes more probable. Since similar energetic situations as for ethylene should be present in a range of molecules, we expect that the dependence of the outcome of fragmentation reactions that involve the breakage of two (or more) chemical bonds on the precise timing of the laser-induced distortions of the electronic system (e.g. by ionization) with respect to concomitant nuclear motion should by no means be special to ethylene.

From the viewpoint of controlling the outcome of quantum dynamics by laser fields, a precise timing of laser-induced modifications of the molecular electronic system with respect to concomitant nuclear motion, as demonstrated here, is the basis for almost any type of molecular control. Coherent quantum control uses weak resonant laser pulse sequences, precisely timed to the nuclear motion, for determining the outcome of molecular dissociation and/or restructuring^28^. Precise timing of a control pulse relative to the motion of the nuclei induced by a pump pulse is also necessary in alternative, strong-field-based control schemes that non-resonantly modify molecular electronic states with an intense light field, e.g., via the Stark-effect^29^.

In the present work only one pulse is used for controlling the outcome of fragmentation reactions, and the nuclear motion and laser-interaction with the electronic system take place within the same pulse. This single-pulse scheme has been successfully applied to control fragmentation of different polyatomic molecules by using the carrier-envelope phase (CEP) of few-cycle pulses as the control parameter^5^,^7^,^9^,^10^. However, even for the short-pulse CEP-control scheme it has been pointed out in studies on 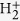 and 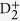 that the dynamics of the nuclei and that of the field-interaction with the electronic system must match, such that the decisive excitation occurs at the optimum nuclear distance^6^,^8^. In the present work the determining nuclear dynamics is the C-H stretch motion. As can be seen in Fig. 2, a pulse duration of at least 12 fs is necessary for reaching the nuclear distance at which two-body fragmentations become enhanced. However, as can also be seen in Fig. 2, in order to achieve matching between the slower C-C stretch dynamics and the decisive electronic excitation (i.e., the third ionization step), the pulses should be much longer than the 25 fs used here.

Our results therefore show that, in order to increase the effectiveness of determining the breakage of a bond that exhibits a slow stretch dynamics, a double-pulse scheme with two precisely timed ∼5 fs pulses is preferred. In such a double-pulse scheme the first pulse populates a PES that exhibits a suitable stretch motion and the second delayed pulse initiates the desired fragmentation by ionization. Experiments that implement this possibility are currently being conducted.

## Methods

### Laser system

Sub-5 fs laser pulses were generated at a repetition rate of 5 kHz by spectral broadening of 25 fs (FWHM) laser pulses from a home-built Titanium-Sapphire laser amplifier system in a gas-capillary and subsequent temporal re-compression with chirped mirrors and a pair of glass wedges. The pulse duration was varied in between 4.5 fs and 17 fs by positively chirping the shortest pulses by propagating them through different amounts of fused silica. Pulses with a duration of 25 fs were used directly from the chirped pulse amplifier system. Further information about the laser setup can be found in our previous publications^5^,^30^.

### Coincidence momentum imaging

The laser beam was focused in an ultra-high vacuum chamber (∼1.3 × 10^−10^ mbar) onto a supersonic gas jet of ethylene molecules using a spherical mirror. The resulting ions were guided to a time and position sensitive channel plate detector by a weak homogeneous electric field (23 V/cm). The polarization direction of the linearly polarized laser beam was along the spectrometer axis. From all ions detected in coincidence, molecular fragmentation channels were selected during the off-line data analysis by applying momentum conservation conditions in all three dimensions. Details about the coincidence selection can be found in Ref. 13.

### Quantum dynamical simulations

The time dependent Schrödinger equation for the nuclear wave packet was solved on a grid with grid spacing *δx* = 0.02 a.u. Propagation in time was accomplished with the Lanczos method (time step size *δt* = 0.25 a.u.), and a mask function was applied at the grid boundary to avoid unphysical reflections. The simulation was started on the PES of 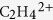 [the lower PES in Fig. 4(a)] with the initial wave function of the form 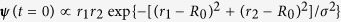, with *R*_0_ = 2 a.u., *σ* = 0.5 a.u. and *r*_1_, *r*_2_ the two C-H distances. This initial state corresponds to instantaneous vertical ionization 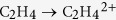 with the nuclear wave function unchanged. The wave function *ψ*(*t*) was propagated on the 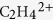 PES until a time *τ*, when the third ionization step is assumed to take place abruptly, and the PES is thus instantaneously changed to the 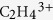 PES. Note, that the influence of the laser field was neglected during the propagation. On the 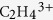 PES, the wave packet eventually dissociates. To separate the contributions from the two-body and the three-body dissociation pathways [green and magenta arrows on the upper PES in Fig. 4(a)], we have integrated the probability flux that was absorbed at the boundary of the grid for two regions: (i) *r*_1_ < *L* or *r*_2_ < *L*, and (ii) both *r*_1_ ≥ *L* and *r*_2_ ≥ *L*, where *L* = 5 a.u. Region (i) corresponds to dissociation along one of the magenta arrows on the upper PES in Fig. 4(a) (two-body dissociation), and region (ii) to dissociation along the green arrow on the upper PES in Fig. 4(a) (three-body dissociation). The sum of the probability in the two channels is one, since all of the wave packet eventually dissociates.

## Additional Information

**How to cite this article**: Xie, X. *et al.* Duration of an intense laser pulse can determine the breakage of multiple chemical bonds. *Sci. Rep.* **5**, 12877; doi: 10.1038/srep12877 (2015).

## Supplementary Material

Supplementary Information
