# Supplementary material for: Duration of an intense laser pulse can determine the breakage of multiple chemical bonds
Source: Sci Rep. 2015 Aug 14;5:12877. doi: 10.1038/srep12877 (PMC4536518; doi:10.1038/srep12877)
Supplement: Supplementary Information [file srep12877-s1.pdf]

Duration of an intense laser pulse can determine the breakage of multiple chemical bonds

Supplementary Information

Xinhua Xie, Erik Lötstedt, Stefan Roither, Markus Schöffler, Daniil Kartashov, Katsumi Midorikawa, Andrius Baltuška, Kaoru Yamanouchi, Markus Kitzler

Analysis of angular momentum distributions

Figs. 1-SM(a) and 1-SM(b) show angular momentum distributions of fragment ions relative to the laser polarization direction (horizontal) for three- and two-body fragmentation reactions, respectively. From the angular distributions it is possible to infer how fast the fragmentation reactions happen in comparison to the rotational motion of the molecular ion.[1-SM, 2-SM, 3-SM]

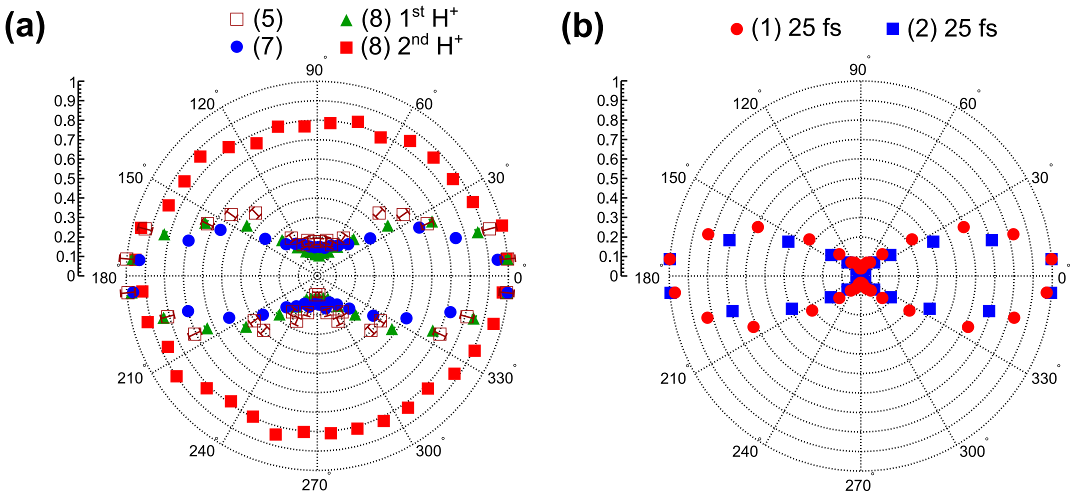

Figure 1-SM: Fragment angular distributions with respect to the laser polarization direction (horizontal). (a) Angular distributions of protons ejected during three-body fragmentations of $C_2H_4^{3+}$ initiated with 25 fs laser pulses. Open dark red squares and blue dots denote the protons ejected during the reactions defined by Eqs. (5) and (7) in the main manuscript, respectively. The green triangles and filled red squares denote the protons ejected during the first and second fragmentation steps of the sequential reaction Eq. (8) of the main manuscript, respectively. (b) Angular distributions of fragments ejected during two-body fragmentations of $C_2H_4^{3+}$ initiated with 25 fs laser pulses for the reactions defined as Eqs. (1) [red dots] and (2) [blue squares] in the main manuscript. The laser peak intensity is $8\times10^{14}\text{W/cm}^2$ for all data points in both panels.

It can be seen that the angular distributions of all fragment ions that are ejected during either the first step of a sequential fragmentation reaction or during a concerted three-body fragmentation reaction strongly peak along the laser polarization direction. The same is true for the two-body fragmentation reactions. The shape of the measured angular distribution of fragment ions can be strongly influenced by molecular alignment initiated by the ionizing laser pulse. It has been shown that this so-called post-ionization alignment (PIA) effect is negligible for few-cycle pulses.[4-SM] We checked by comparing the angular momentum distributions measured for laser pulse durations of 4.5 and 25 fs that the anisotropic angular momentum distributions in Fig. 1-SM(a) are not affected by PIA owing to the fast fragmentation of the triply charged molecular ion. The strongly anisotropic shapes therefore show that these fragmen-

tation reactions take place fast as compared to the rotational period of ethylene about the C-C axis.[1-SM] The rotational period can be calculated from the rotational constant. For the symmetry of ethylene there exist two rotational constants for rotation about the C-C axis, which have been measured to be just slightly different, namely 1 and 0.83 cm^{-1}, respectively.[5-SM] From these values the two rotational periods are calculated to be approximately 17 and 20 ps. Thus, the fragmentation reactions discussed above take place much faster than 17 ps. In contrast, the isotropic angular distribution of the proton ejected during the second step of the sequential three-body fragmentation reaction, Eq. (8) in the main manuscript, shown by red squares in Fig. **1-SM**(a), indicates that the second step takes place on a time-scale comparable or longer than the rotational period. In any case the second fragmentation step takes place much later than the first one. Thus, the dications formed during the first fragmentation step of this channel are metastable and exhibit a long lifetime in the tens of picoseconds range, which is much longer than the vibrational dynamics of the C-H and C-C bonds. In contrast, the very directed ejection of the moieties along the laser polarization direction during the first fragmentation step indicates that no meta-stable ethylene trication exists. After the removal of three electrons the molecular trication dissociates into two or three fragments on a time scale that is much faster as compared to the rotational period. Therefore, the angular distributions in Fig. **1-SM** directly reflect the fragmentation direction of the first fragmentation step. This finding opens up the possibility to interpret them with respect to the underlying angular dependence of the ionization probability that is determined by the shape of the molecular orbitals that are involved in the ionization,[6-SM, 7-SM, 8-SM, 9-SM, 10-SM, 11-SM, 12-SM, 13-SM] as described in the main manuscript.

References

1-SM Hishikawa, A., Hasegawa, H. & Yamanouchi, K. Hydrogen Migration in Acetonitrile in Intense Laser Fields Studied by Coincidence Momentum Imaging. *J. Electron Spectros. Relat. Phenomena* **141**, 195–200 (2004).

2-SM Okino, T. *et al.* Coincidence momentum imaging of ejection of hydrogen molecular ions from methanol in intense laser fields. *Chem. Phys. Lett.* **419**, 223–227 (2006).

3-SM Zhang, L. *et al.* Path-selective investigation of intense laser-pulse-induced fragmentation dynamics in triply charged 1,3-butadiene. *J. Phys. B* **45**, 085603 (2012).

4-SM Tong, X. M. *et al.* Post ionization alignment of the fragmentation of molecules in an ultrashort intense laser field. *J. Phys. B* **38**, 333–341 (2005).

5-SM Herzberg, G. *Electronic spectra and electronic structure of polyatomic molecules* (Van Nostrand, New York, 1966).

6-SM Xie, X. *et al.* Electronic Predetermination of Ethylene Fragmentation Dynamics. *Phys. Rev. X* **4**, 021005 (2014).

7-SM Xie, X. *et al.* Selective Control over Fragmentation Reactions in Polyatomic Molecules Using Impulsive Laser Alignment. *Phys. Rev. Lett.* **112**, 163003 (2014).

8-SM Hosaka, K., Itakura, R., Yokoyama, K., Yamanouchi, K. & Yokoyama, A. Photoelectron-photoion coincidence momentum imaging for dissociative ionization of ethanol in intense laser fields. *Chem. Phys. Lett.* **475**, 19–23 (2009).

9-SM Alnaser, A. *et al.* Effects Of Molecular Structure on Ion Disintegration Patterns In Ionization of O_2 and N_2 by Short Laser Pulses. *Phys. Rev. Lett.* **93**, 113003 (2004).

10-SM Tong, X., Zhao, Z. & Lin, C. Theory of molecular tunneling ionization. *Phys. Rev. A* **66**, 033402 (2002).

11-SM Muth-Böhm, J., Becker, A. & Faisal, F. Suppressed Molecular Ionization for a Class of Diatomics in Intense Femtosecond Laser Fields. *Phys. Rev. Lett.* **85**, 2280–2283 (2000).

12-SM Talebpour, A., Bandrauk, A., Yang, J. & Chin, S. Multiphoton ionization of inner-valence electrons and fragmentation of ethylene in an intense Ti:sapphire laser pulse. *Chem. Phys. Lett.* **313**, 789–794 (1999).

13-SM Wu, C. *et al.* Tunneling ionization of carbon dioxide from lower-lying orbitals. *Phys. Rev. A* **83**, 033410 (2011).
